# A Case of Spontaneous Regression of Primary Tumor after Adrenalectomy for Primary Lung Cancer with Synchronous Adrenal Metastasis

**DOI:** 10.70352/scrj.cr.25-0379

**Published:** 2025-08-13

**Authors:** Hisaya Chikaraishi, Ryu Kanzaki, Hironobu Samejima, Masao Kobayashi, Julian Horiguchi, Tomohiro Maniwa, Hidetoshi Satomi, Keiichiro Honma, Jiro Okami

**Affiliations:** 1 Department of General Thoracic Surgery, Osaka International Cancer Institute, Osaka, Osaka, Japan; 2 Department of Diagnostic Pathology, Osaka International Cancer Institute, Osaka, Osaka, Japan

**Keywords:** adrenal metastases, oligometastases, spontaneous regression

## Abstract

**INTRODUCTION:**

Adrenal metastasis from primary lung cancer is relatively common, occurring in approximately 5%–10% of clinical cases. Long-term survival can be achieved through surgical resection of adrenal metastases in addition to primary lesions. Spontaneous regression of cancer is defined as the partial shrinkage or complete disappearance of cancer following no treatment or treatment considered ineffective against cancer. Spontaneous regression of non-small cell lung cancer (NSCLC) is rare. Here, we describe a case of NSCLC with adrenal metastasis, in which the primary tumor exhibited spontaneous regression and was pathologically absent following surgical treatment of the metastatic lesion.

**CASE PRESENTATION:**

A 59-year-old male patient was referred to our department with elevated carcinoembryonic antigen levels and an abnormal opacity on chest CT. Contrast-enhanced CT revealed a 2-cm lesion in the right upper lobe of the lung and a 4.5-cm mass in the right adrenal gland. Bronchoscopic biopsy confirmed non-small cell carcinoma (cT1bN0M1b, cStage IVA). As the adrenal metastasis was considered oligometastatic based on ^18^F-fluorodeoxyglucose PET/CT and head MRI, surgical resection of both the primary lung lesion and the adrenal metastasis was planned. Laparoscopic right adrenalectomy was performed, and histological examination confirmed adrenal metastasis from lung cancer. Postoperatively, no new metastases were detected, and CT demonstrated a reduction in the size of the primary lesion. Robot-assisted right upper lobectomy with lobe-specific nodal dissection was subsequently performed. Pathological examination revealed no malignant findings in the resected right upper lobe. The patient was discharged without complications and remains recurrence-free 5 months after surgery.

**CONCLUSIONS:**

This report presents a case of primary lung cancer with adrenal metastasis in which the primary tumor underwent spontaneous regression and was pathologically absent following surgical treatment of the metastasis.

## Abbreviations

ALK
: anaplastic lymphoma kinase
BRAF
: B-rapidly accelerated fibrosarcoma factor
CD4
: cluster of differentiation 4
CD8
: cluster of differentiation 8
CEA
: carcinoembryonic antigen
CK19
: cytokeratin 19
CK7
: cytokeratin 7
EGFR
: epidermal growth factor receptor
FDG
: ^18^F-fluorodeoxyglucose
HER2
: human epidermal growth factor receptor 2
MET
: mesenchymal–epithelial transition
NSCLC
: non-small cell lung cancer
PD-L1
: programmed cell death ligand 1
RET
: rearranged during transfection
ROS1
: c-ros oncogene 1, receptor tyrosine kinase
SF-1
: steroidogenic factor 1
SUVmax
: maximal value of standardized uptake value
TPS
: tumor proportion score

## INTRODUCTION

Adrenal metastases are recognized manifestations of advanced lung cancer and may occasionally present as a solitary lesion.^1)^ In carefully selected patients with a limited metastatic burden—referred to as oligometastatic disease—aggressive local treatment, including surgical resection, may be considered.^2–6)^ Spontaneous regression of malignancy, defined as partial or complete tumor reduction in the absence of effective therapy, is rare, particularly in NSCLC.^7,8)^ Although isolated reports have been documented, cases with pathological confirmation of complete tumor disappearance are exceedingly uncommon. This report describes a rare case of NSCLC with synchronous adrenal metastases in which the primary tumor underwent spontaneous regression, ultimately culminating in pathological disappearance following adrenalectomy.

## CASE PRESENTATION

**Fig. 1:**
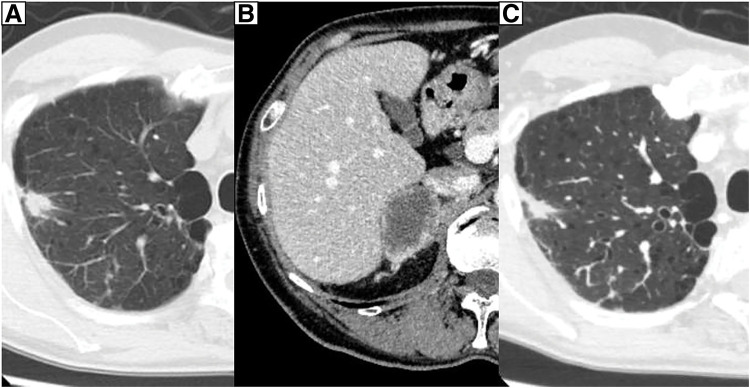
Contrast-enhanced CT of the thorax and abdomen. CT shows an irregular 2-cm-sized nodular shadow with spicules just below the dorsal segment (S2) pleura of the right upper lobe (**A**), and a 4.5-cm mass was observed in the right adrenal gland (**B**). On CT following adrenalectomy, the perpendicular diameter of the primary lesion had decreased to 1.0 cm (**C**).

**Fig. 2:**
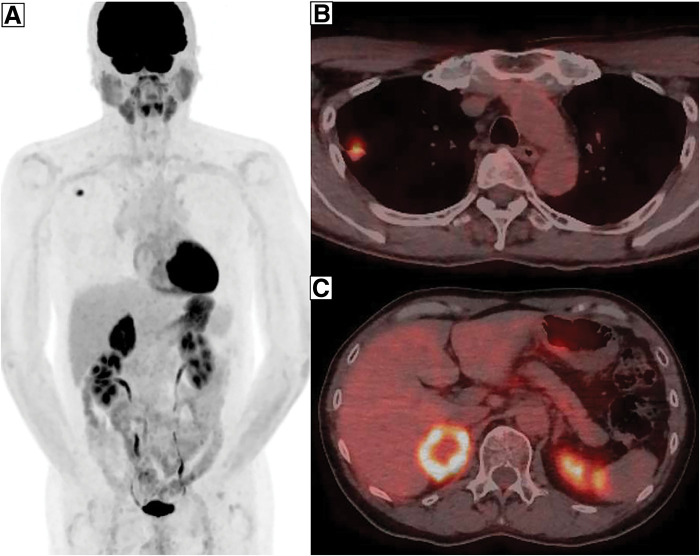
FDG-PET scans. FDG-PET scans show abnormal uptake (**A**) in the lung nodule (**B**, SUVmax = 7.2) and in the adrenal mass (**C**, SUVmax = 11.6). FDG, ^18^F-fluorodeoxyglucose; SUVmax, maximal value of standardized uptake value

**Fig. 3:**
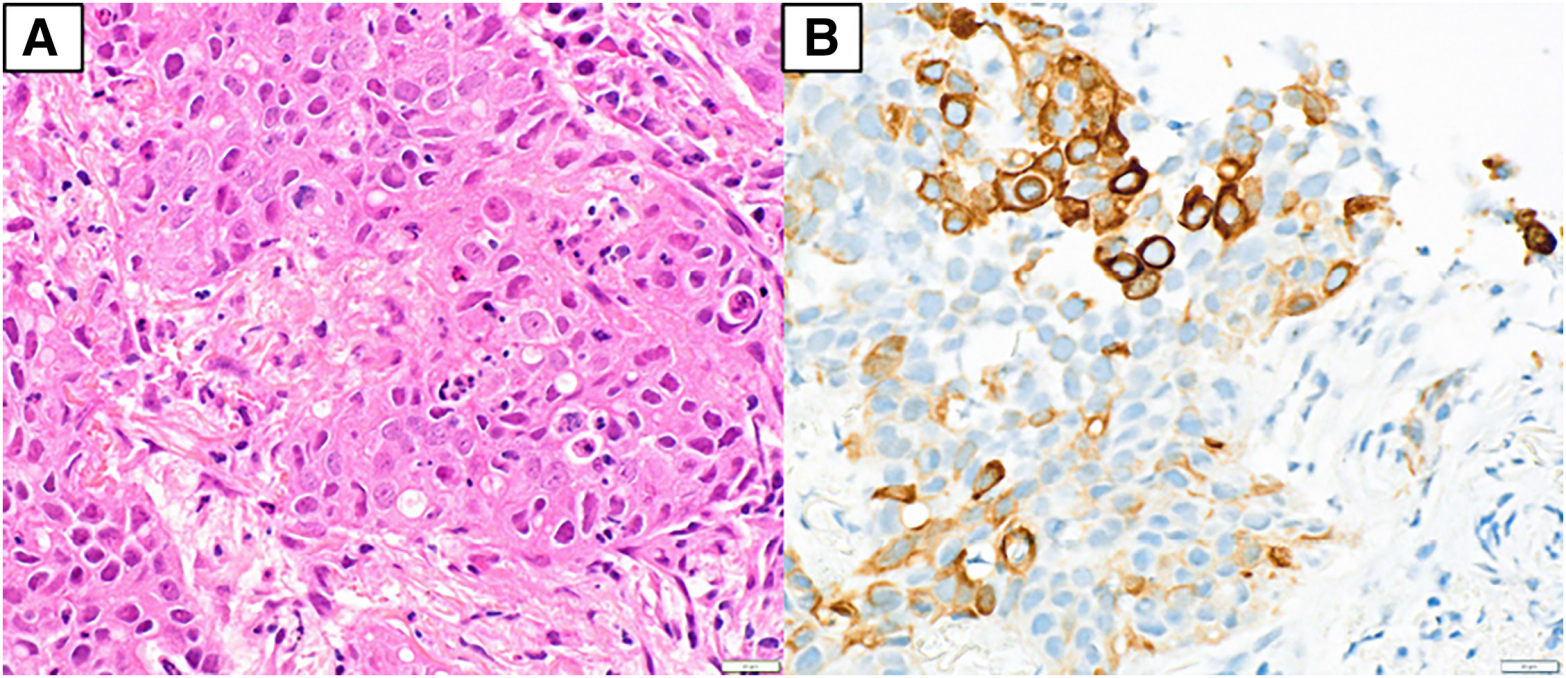
Bronchoscopic biopsy. The specimen from the bronchoscopic biopsy shows that the nodule of the right lung appears to be NSCLC (**A**, hematoxylin and eosin stain, ×400 original magnification), and the cells were positive for CK7 (**B**) (×400). CK7, cytokeratin 7; NSCLC, non-small cell lung cancer

On March X, a 59-year-old male patient was referred to the department due to elevated serum CEA levels identified during a routine medical checkup. The patient had no significant past medical history and no regular use of supplements or medications. Blood count and biochemical analyses revealed no abnormalities. Tumor marker testing showed elevated CEA (128.3 ng/mL) and CK19 fragment (5.3 ng/mL) levels. The levels of progastrin-releasing peptide, squamous cell carcinoma-associated antigen, and neuron-specific enolase were within normal limits. Chest radiography showed no obvious abnormalities. Contrast-enhanced CT of the thorax and abdomen identified a 2.0-cm irregular nodular lesion with spiculated margins located just beneath the pleura of the dorsal segment (S2) of the right upper lobe. The tumor measured 1.5 cm in perpendicular diameter on CT scan (**Fig. 1A**). No apparent lymphadenopathy was noted. Additionally, a 4.5-cm mass was detected in the right adrenal gland (**Fig. 1B**). FDG-PET/CT demonstrated increased uptake in the lung lesion, with an SUVmax of 7.2 (**Fig. 2A** and **2B**). Hypermetabolic activity was also observed in the adrenal mass, with an SUVmax of 11.6 (**Fig. 2A** and **2C**). MRI of the head showed no evidence of brain metastases. A bronchoscopic biopsy was performed, and a neoplastic lesion was suspected. However, the atypia of the clustered cells was relatively mild, and neither irregular keratinization nor well-defined glandular structures were observed. Therefore, immunohistochemical evaluation was required to establish a definitive diagnosis of adenocarcinoma. Careful differentiation from benign lesions, such as inflammatory pseudotumors or infectious diseases, was also necessary. Based on the cellular morphology and positive immunostaining for CK7—which is known to be positive in primary lung cancer but negative in primary adrenal tumors (**Fig. 3A** and **3B**)—the lesion was diagnosed as non-small cell carcinoma. In addition, the negativity of SF-1 and Melan-A, which are often positive in primary adrenal tumors, also supported the conclusion that the adrenal lesion was a metastatic tumor. Immunohistochemical analysis revealed a PD-L1 TPS of 10%, with no clear histological features indicative of poor differentiation. These findings led to a diagnosis of NSCLC (cT1bN0M1b, cStage IVA). Given the presence of a solitary adrenal metastasis, the disease was considered oligometastatic. Therefore, surgical resection of the adrenal mass was prioritized for definitive diagnosis and biomarker evaluation, with resection of the primary tumor planned subsequently if no further metastases were identified during follow-up.

**Fig. 4:**
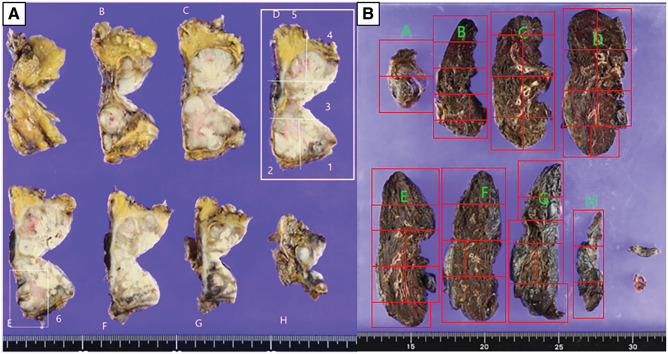
Photographs of surgical specimens. Adrenal gland (**A**) and the right upper lobe (**B**).

**Fig. 5:**
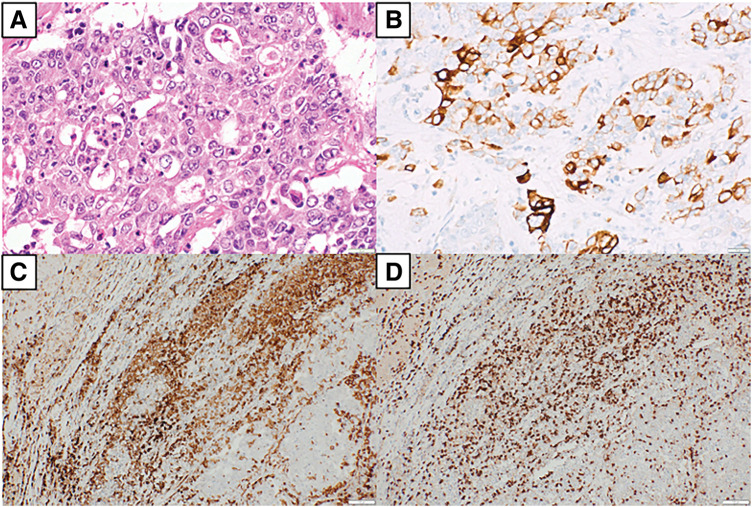
Histological findings. (**A**) Lesion of the adrenal mass appears to be adrenal metastasis of NSCLC (hematoxylin and eosin stain, ×400 original magnification), and the cells were positive for CK7 (**B**) (×400). It shows inflammatory cell infiltration, and the cells were positive for CD4 (**C**) and CD8 (**D**) (×100). CD4, cluster of differentiation 4; CD8, cluster of differentiation 8; CK7, cytokeratin 7; NSCLC, non-small cell lung cancer

On May X, 34 days after bronchoscopic biopsy, a laparoscopic right adrenalectomy was performed (**Fig. 4A**). The size of the resected specimen was generally consistent with the preoperative CT findings. Histopathological analysis confirmed adrenal metastasis originating from NSCLC, based on morphological similarity and consistent CK7 immunoreactivity between the adrenal specimen and the bronchoscopic biopsy of the lung lesion (**Fig. 5A** and **5B**). Infiltration by inflammatory cells was noted, with immunohistochemistry demonstrating the presence of CD4- and CD8-positive lymphocytes (**Fig. 5C** and **5D**). Biomarker testing using the Oncomine Dx Target Test Multi-CDx System (Thermo Fisher Scientific, Waltham, MA, USA) revealed no detectable mutations in BRAF, EGFR, or HER2. The test results for ALK, ROS1, RET, and MET were inconclusive. Immunohistochemical analysis revealed a PD-L1 TPS of 10%, as well as an absence of histological features suggestive of poor differentiation, both consistent with the findings in the primary tumor. The CEA level measured at 5 weeks after adrenalectomy showed a marked decrease to 10.0 ng/mL, although it remained mildly elevated. Additionally, on CT following adrenalectomy, the maximum diameter of the primary lesion remained unchanged; however, the perpendicular diameter showed a slight reduction to 1.0 cm (**Fig. 1C**). As no new metastases were identified following adrenal resection, surgical removal of the primary lung tumor was carried out as planned.

### Operative findings

On August X, 78 days after adrenalectomy, robot-assisted right upper lobectomy was performed. Upper mediastinal node dissection followed the upper lobe resection, and the procedure was completed successfully. The operation duration was 316 min, and blood loss was minimal.

### Histopathological findings

**Fig. 6:**
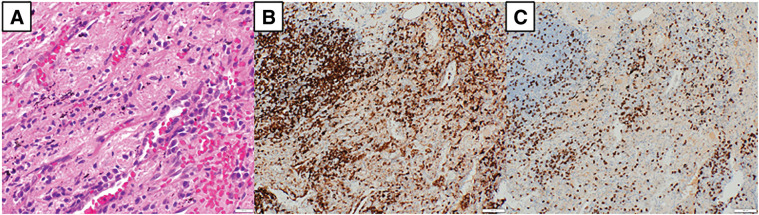
Histological findings. (**A**) Lesion of the lung shows no malignant cells but is infiltrated by inflammatory cells (hematoxylin and eosin stain, ×400 original magnification), and the cells were positive for CD4 (**B**) and CD8 (**C**) (×100). CD4, cluster of differentiation 4; CD8, cluster of differentiation 8

Specimens were evenly sectioned, and all sections underwent sampling. To assess lesion localization as anticipated preoperatively (**Fig. 4B**), additional deep sectioning was performed near the lesion, including re-embedding to prepare sections from the specimen’s reverse side and further processing of remaining tissue as appropriate. The nodule suspected as the primary tumor corresponded to nodular atelectasis with calcification and anthracosis. No pathological evidence of malignancy was identified (**Fig. 6A**). Inflammatory cell infiltration was present in the same area; immunostaining revealed predominance of CD4-positive lymphocytes alongside CD8-positive lymphocytes (**Fig. 6B** and **6C**). The extent of inflammatory cell infiltration was comparable to that observed in the adrenal metastatic lesion.

### Postoperative clinical course

The final diagnosis was NSCLC, pT0N0M1b, pStage IVA. The postoperative course was uneventful, and the patient was discharged without complications. Regarding postoperative adjuvant therapy, given the insufficient evidence supporting adjuvant treatment for oligometastatic disease, the patient was thoroughly informed of the potential benefits and risks. Based on this discussion and the patient’s preference, adjuvant therapy was not administered. The CEA level has normalized, and no evidence of recurrence or metastasis has been observed. The patient remains alive and recurrence-free at 8 months following lung resection.

## DISCUSSION

Adrenal metastasis from primary lung cancer is common, followed by lung, liver, bone, and brain, with a frequency of 5%–10% in clinical cases.^1)^ Chemotherapy is usually administered to patients with NSCLC and distant metastases. The European consensus defines synchronous oligometastatic NSCLC as up to 5 metastases in no more than 3 organs, with mandatory staging using FDG-PET/CT and brain MRI or CT, and eligibility for radical treatment with acceptable toxicity.^9)^ Several reports have suggested that when distant metastases are deemed oligometastatic, local treatment of both the primary tumor and metastatic sites, including adrenalectomy, can improve long-term outcomes.^2–4)^ In particular, it has been reported that resection of adrenal metastases on the same side as the primary tumor has a better prognosis than resection of adrenal metastases on the opposite side. This is because metastasis from the lung to the ipsilateral adrenal gland is not hematogenous but is localized by lymphatic flow in the retroperitoneal route.^5,6)^ These reports suggest that resection of both the primary lung tumor and the adrenal metastases may offer long-term recurrence-free survival. In the present case, the tumor was diagnosed as an adrenal metastasis of lung cancer based on the morphological features of the tumor cells and immunoreactivity for CK7, SF-1 and Melan-A. Accordingly, a favorable prognosis was anticipated in this patient.

According to Cole and Everson, spontaneous regression of cancer is a pathological condition in which cancer shrinks or disappears spontaneously without or with treatment that is not supposed to be effective against cancer.^7)^ Spontaneous regression of NSCLC is considered to be extremely rare.^8)^ Since 2011, there have been 8 reported cases of surgical treatment for primary NSCLC that showed spontaneous regression (**Table 1**).^10–17)^ Seven of these cases were reported in Japan, consistent with previous reports showing that spontaneous regression of primary NSCLC is relatively common in Japan.^18)^ Mizuno et al. reported the first case of complete pathological disappearance due to spontaneous regression,^10)^ and only 2 cases have since been reported.^15,16)^ This is the 4th reported case of pathologically proven complete disappearance of NSCLC due to spontaneous regression. None of the 3 previously reported cases involved distant metastasis, and this is the first case in which complete disappearance due to spontaneous regression of primary NSCLC with distant metastasis was pathologically proven.

**Table 1:** Cases of spontaneous regression with surgery of NSCLC

	Author	Year	Age/gender	Histology	c-Stage	p-Stage	Cause of spontaneous regression
1	Mizuno et al.^10)^	2011	62/Male	NSCLC	cT1aN0M0	pT0N0M0	Biopsy
2	Furukawa et al.^11)^	2011	56/Male	SCC	N/A	pT1N0M0	Uncertain
3	Tomizawa et al.^12)^	2015	85/Female	LCNEC	cT1cN0M0	pT4N0M0	Uncertain
4	Matsui et al.^13)^	2018	56/Female	SCC	cT1bN2M0	pT1aN2M0	Biopsy
5	Uchida et al.^14)^	2019	20/Female	N/A	cT1bN0M0	pT1bN0M0	Uncertain
6	Yamamoto et al.^15)^	2022	69/Male	SCC	N/A	pT0N0M0	Biopsy
7	Schiavon et al.^16)^	2022	74/Male	Adeno	cT2aN0M0	pT0N0M0	Aspiration pneumonia or biopsy
8	Koike et al.^17)^	2024	76/Male	SCC	cT1bN0M0	pT1bN0M0	Uncertain
9	Present case	2025	59/Male	NSCLC	cT1bN0M1b	pT0N0M0	Biopsy or surgery for metastases

Adeno, adenocarcinoma; LCNEC, large-cell neuroendocrine carcinoma; N/A, not applicable; NSCLC, non-small cell lung cancer; SCC, squamous cell carcinoma

To assess the therapeutic efficacy of adrenalectomy, we monitored the perioperative changes in serum CEA levels. The serum half-life of CEA is generally estimated to be approximately 3–7 days. In patients with preserved hepatic function, CEA levels are expected to decrease to near-normal levels within 4–6 weeks postoperatively, corresponding to approximately 5 half-lives.^19)^ In the present case, liver function was preserved, suggesting that any CEA changes attributable to adrenalectomy would be reliably reflected by 6 weeks postoperatively. However, in this case, CEA was measured approximately 5 weeks after adrenalectomy. Therefore, the elevated preoperative serum CEA level was considered to have been primarily attributable to the metastatic lesions in the adrenal gland.

Various hypotheses have been proposed regarding the mechanisms of spontaneous regression. Clinically, it is often triggered by surgery, invasive procedures, or infections, suggesting immune activation against tissue invasion.^20,21)^ Infiltration of CD8- and CD4-positive T cells into tumor tissue has been implicated in tumor shrinkage.^22–24)^ In our case, both cell types were observed in the primary lung tumor and adrenal metastases, suggesting that the immune response may have played a key role. To understand the involvement of the immune response in tumor regression, it is important to assess the degree of infiltration by CD4- and CD8-positive lymphocytes. In the present case, although quantitative or semi-quantitative evaluation was challenging due to the presence of necrotic tissue in the adrenal lesion and marked, heterogeneous fibrosis in the primary tumor, no substantial difference in the degree of lymphocyte infiltration was observed between the 2 lesions. It remains unclear whether these lymphocytes are tumor-specific, and further case studies are needed to elucidate this mechanism. Previous reports have suggested that spontaneous regression in NSCLC may be associated with high PD-L1 expression and poorly differentiated histological features.^25)^ However, in the present case, PD-L1 expression was 10% in both lesions, and there were no definitive histopathological findings indicative of poor differentiation. Further accumulation of cases is necessary to establish a clearer understanding of the relationship between PD-L1 expression, tumor differentiation, and spontaneous regression. In our case, adrenalectomy and bronchoscopic biopsy may activate the immune system. In previous reports, biopsy of the metastases caused spontaneous regression of the primary tumor,^26,27)^ and adrenalectomy may have caused the spontaneous regression in this case. However, the presence of inflammatory cell infiltration in the metastases suggests that the bronchoscopic biopsy performed prior to adrenalectomy may have caused spontaneous regression.

Of the primary and metastatic lesions, both of which showed inflammatory cell infiltration, only the primary lesion showed spontaneous regression, suggesting that, in addition to the mechanism proposed above, other factors may have contributed to the spontaneous regression in this case. The relationship between metastatic and primary tumors is bidirectional, and the presence of metastatic tumors causes primary tumors to grow and further disseminate.^28)^ Therefore, in our case, adrenalectomy performed during the ongoing spontaneous regression triggered by bronchoscopic biopsy may have further enhanced the regression by boosting the immune response and removing metastatic support from the primary tumor. Evidence supporting local therapy for oligometastatic disease remains limited, and the concomitant use of systemic chemotherapy should be carefully considered. Although chemotherapy was not administered in the present case, close follow-up is warranted.

### Limitation

Reporting cases of spontaneous regression of cancer inherently involves certain limitations. One of the main concerns is the possibility of overdiagnosis at the initial evaluation, particularly in small lesions, as well as the potential for misdiagnosis of benign conditions such as inflammatory pseudotumors or infectious lesions. In the present case, the diagnosis of malignancy was confirmed by histopathological examination of a bronchoscopic biopsy specimen, with immunohistochemical findings supporting the diagnosis of non-small cell lung carcinoma. Furthermore, comprehensive clinical, radiological, and pathological evaluations were conducted to support the diagnosis of spontaneous regression. These efforts aimed to minimize the likelihood of misdiagnosis and ensure the validity of the observed spontaneous regression.

## CONCLUSIONS

This report highlights a case of primary lung cancer with adrenal metastasis in which the primary tumor showed spontaneous regression and completely disappeared after surgical treatment of the metastasis.

## ACKNOWLEDGMENTS

The authors thank Editage (https://www.editage.jp) for the English language review of the manuscript.

## DECLARATIONS

### Funding

Not applicable.

### Authors’ contributions

H. Chikaraishi analyzed and interpreted the patient data and was a major contributor to writing the manuscript.

All the authors have read and approved the final version of the manuscript.

### Availability of data and materials

Not applicable.

### Ethics approval and consent to participate

This work does not require ethical considerations or approval. Informed consent to participate in this study was obtained from the patient.

### Consent for publication

Written informed consent was obtained from the patient for publication of this report and its accompanying images.

### Competing interests

The authors have no competing interests associated with this manuscript.
